# Cannabinoid Hyperemesis Syndrome in Adolescents: A Narrative Review

**DOI:** 10.3390/pediatric17040075

**Published:** 2025-07-14

**Authors:** Camilla Pietrantoni, Gaia Margiotta, Giuseppe Marano, Marianna Mazza, Francesco Proli, Giuseppe Stella, Alessia Cherubino, Francesca Viozzi, Fabiana Rita Guida, Claudia Rendeli, Roberto Pola, Eleonora Gaetani, Valentina Giorgio

**Affiliations:** 1 Unità Operativa Semplice Dipartimentale Spina Bifida e Uropatie Congenite, Fondazione Policlinico Universitario “A. Gemelli” IRCCS, Università Cattolica del Sacro Cuore, 00168 Rome, Italy; 2 Unit of Psychiatry, Fondazione Policlinico Universitario “A. Gemelli” IRCCS, 00168 Rome, Italy; mariannamazza@hotmail.com; 3 Department of Neurosciences, Università Cattolica del Sacro Cuore, 00168 Rome, Italy; 4 Section of Internal Medicine and Thromboembolic Diseases, Department of Internal Medicine, Fondazione Policlinico Universitario “A. Gemelli” IRCCS, Università Cattolica del Sacro Cuore, 00168 Rome, Italy; 5 Department of Translational Medicine and Surgery, Fondazione Policlinico Universitario “A. Gemelli” IRCCS Università Cattolica del Sacro Cuore, 00168 Rome, Italy; eleonora.gaetani@unicatt.it; 6 Unit of Internal Medicine, Cristo Re Hospital, 00167 Rome, Italy

**Keywords:** cannabinoid hyperemesis syndrome, adolescents, cyclic vomiting

## Abstract

Cannabinoid hyperemesis syndrome (CHS) is characterized by a pattern of cyclic vomiting and abdominal pain despite an absence of an organic cause, occurring in regular cannabis users. This syndrome was first described in 2004. Initially considered rare, with the increased use and legalization of cannabis, a growing incidence of diagnoses has been observed. Data on the pediatric population are still scant despite the high rate of cannabis consumption in young people. In this narrative review, we aim to synthesize the growing knowledge about CHS and its epidemiology, pathophysiology, diagnosis, and management in the pediatric population. Findings in this review highlight the diagnostic challenges in pediatric patients, the limited efficacy of standard anti-emetic therapies, and the central role of cannabis cessation in treatment. This review underscores the need for increased awareness of CHS in pediatric practice to ensure timely diagnosis and avoid unnecessary investigations and interventions.

## 1. Introduction

### 1.1. Definition and Brief History of Cannabinoid Hyperemesis Syndrome (CHS)

Cannabinoid hyperemesis syndrome (CHS) is a condition that leads to repeated and severe bouts of vomiting as a consequence of the long-term use of marijuana [1]. CHS shares many clinical features with Cyclic Vomit Syndrome (CVS) except for its association with chronic cannabis use.

In fact, CHS occurs in some individuals who use cannabis chronically and is characterized by recurrent episodes of severe nausea, vomiting, and abdominal pain. A hallmark of CHS is the symptomatic relief achieved through hot showers or baths. It was first described by Allen et al. in 2004 [2]. Since then, numerous studies and case reports have discussed this condition, describing its pathophysiology, clinical features, and treatment modalities. The criteria used to define CHS have varied widely across the literature. This inconsistency has fueled significant debate and uncertainty regarding the classification of CHS as a distinct clinical condition from CVS [3].

The first diagnostic criteria for CHS were identified in 2009 by Sontineni et al. [4]. Then, in 2012, a case series involving 98 patients was published by the Mayo Clinic, revising and expanding the diagnostic criteria to include severe cyclic nausea and vomiting, abdominal pain, weekly marijuana use, symptom relief with hot showers or baths, and resolution with cannabis cessation as major criteria [5].

In 2016, the Rome IV criteria, currently the most widely used for diagnosing CHS, were established. However, these criteria were developed using data from the adult population. These diagnostic criteria required the resolution of vomiting episodes after prolonged abstinence from cannabis, although the exact timeframe for symptom resolution was not clearly defined. The characteristic behavior of “hot water bathing” was considered a supporting criterion, despite that, actually, it also occurs in approximately 50% of patients with CVS who do not use cannabis [3].

The first pediatric-specific criteria were only described in 2020 by Lonsdale H et al. Their study found that only 3 of the 34 patients with CHS included in their study would meet the Rome IV criteria for CHS diagnosis in adults, suggesting that these criteria may not be suitable for adolescents [6].

### 1.2. Cannabinoid Use Among Adolescents and Young Adults

Cannabis is one of the most commonly used psychoactive substances in developed countries, with its consumption see a steady increase since the mid-20th century. In particular, in developed regions such as North America, Western Europe, and Australia, cannabis has become closely linked to youth culture, with the initiation age being generally lower than that of other drugs [7]. The increase in consumption among young adults may be related to the perceived reduced risk associated with cannabis use [8]. In the European context, cannabis consumption is more common among people aged 15 to 24 years, with a daily or almost daily use in 2.0% of this age range population [9]. In the United States, 4.9% of adolescents reported having tried marijuana for the first time before the age of 13, and 15.8% reported that they were currently using marijuana (at least once in the 30 days prior to the survey) [10]. Currently, the prevalence of cannabis use among adolescents is higher than that of adults globally (5.5 per cent compared with 4.4 per cent, respectively), particularly in Oceania and Europe [11].

Regarding trends in cannabis use over the years, national surveys reveal heterogeneous patterns. Some countries report increases, others stability, and still others decrease [9]. At the same time, the increase in cannabis use among parents with children at home—from 4.9% to 6.8% between 2002 and 2015—suggests a rise in domestic availability. A relevant factor is risk perception: despite potential harms, only 24% of adolescents aged 12 to 17 perceive cannabis use as risky. Furthermore, the emergence of electronic cigarette devices facilitates the covert consumption of THC [8]. The availability of cannabis-based products is increasingly diverse, including edibles, high-potency products, and various derivatives [9].

Legislative approaches to cannabis vary across countries; some have legalized home cultivation or non-profit cannabis clubs, while others have increased penalties.

Research suggests that restrictive interventions may be associated with a decrease in the overall prevalence of cannabis use and in perceived availability among adolescents, particularly among occasional users. In contrast, more liberal reforms appear to be linked to an increase in overall or experimental use among adolescents [12].

Cannabis also has therapeutic indications in the field of pediatrics; for example, it is used for treating drug-resistant epilepsy (with particular attention to syndromes such as Lennox–Gastaut, Dravet, tuberous sclerosis complex, and fragile X syndrome), chemotherapy-induced nausea and vomiting, autism spectrum disorder, as well as in traumatic brain injury and spasticity [13].

At the same time the use of cannabinoids is associated with acute and chronic adverse effects, while direct mortality is considered rare. Acutely, it can lead to euphoria, mood changes, fatigue, dizziness, and decreased concentration [13] as well as more severe effects such as lethargy, sedation, ataxia, seizures, respiratory depression, and myocarditis [8]. Chronic use, on the other hand, is linked to the development of cognitive impairment and brain damage [14]. In recent decades, it has also been found to be associated with CHS [8].

### 1.3. Purposes of the Review

This review aims to provide a comprehensive summary of current global knowledge on CHS in adolescents, including its epidemiology, diagnosis, pathogenesis, and management.

## 2. Materials and Methods

A literature search on the PubMed/Medline library was performed, using the words “cannabinoid hyperemesis syndrome” and filters for age (from 0 to 18 years). Articles published from 2010 to 2024 were included. English language restriction was applied while geographical restrictions were not. No restrictions on sex were made or other restrictions were made. A total of 47 articles were initially identified. Additionally, references of extracted articles were manually searched. In the end, because of their relevance to the pediatric population and clinical focus, a total of 24 articles were included.

## 3. Results

### 3.1. Epidemiology

The exact incidence and prevalence of CHS among pediatric cannabis users remain unknown [15].

CHS has an estimated incidence of 0.1% in adults in Western countries. Although marijuana use is increasing, there are few pediatric studies on CHS, making it difficult to estimate its incidence in the adolescent population [16].

A possible explanation for the emergence of CHS in recent years is the increase in the tetrahydrocannabinol (THC) average concentration in cannabis, which rose from 4% in 1995 to 12% in 2014. During the same period, the THC-to-cannabidiol ratio also shifted significantly from 14:1 to 80:1 [15]. Given the increased demand for high-potency marijuana, the cannabis user and producer community is selecting and cultivating plants to achieve a higher THC content. This has led to a shift in the production of illicit cannabis plant material, moving from regular marijuana to sinsemilla. Sinsemilla is significantly more potent than common marijuana [17].

In the pediatric population, CHS appears to be more common among females, with the youngest reported case involving a 13-year-old patient [6]. Additionally, a substantial proportion (21%) of adolescents diagnosed with CHS have a history of anxiety and depression [1].

Regarding the toxicological history, according to a 10-year pediatric case series, patients with CHS had a history of cannabis use for a median duration of 42 months (range: 3–93) prior to symptom onset, with a median usage frequency of 21 times per week [6].

### 3.2. Pathophysiology

The pathophysiology of CHS is not yet fully understood [15]. Several hypotheses have been proposed, with the most widely recognized being the dysregulation of the endocannabinoid system. The endocannabinoid system is involved in numerous physiological processes, including gastrointestinal motility, nausea and vomiting, appetite regulation, inflammation, pain modulation, and other functions. The literature indicates that cannabinoid receptor agonists inhibit vomiting, while antagonists may trigger or intensify it. Thus, the endocannabinoid system plays a key role in regulating hyperemesis syndromes, including CHS and cyclic vomiting syndrome.

The cannabinoid receptors CB1R and CB2R are located in the central and peripheral nervous systems, specifically at the presynaptic terminals of excitatory and inhibitory neurons. CB1R is particularly abundant in the dorsal vagal complex, a critical region for emesis. Endogenous ligands are synthesized on demand to regulate stress, nausea, and vomiting, while antagonists can initiate or exacerbate emesis [3]. Additionally, CB1Rs are found in the enteric plexuses. The activation of these plexuses by THC may reduce peristalsis and intestinal secretion, slowing gastric emptying and resulting in pain, nausea, and vomiting. The pro-emetic effects of cannabis in the enteric system may override its central anti-emetic effects, promoting emesis [18]. The role of CB2R in nausea and vomiting is less clear.

Cannabinoids exhibit a biphasic mechanism of action, with anti-emetic effects at low doses and pro-emetic effects at high doses. Marijuana leaves contain over 400 chemical compounds, 60 of which have a cannabinoid structure characterized by a long half-life and lipophilicity. These substances can accumulate in the brain, leading to prolonged exposure [15] and, consequently, to a pro-emetic effect.

Another hypothesis concerns the effects of chronic cannabis use on the hypothalamic–pituitary–adrenal (HPA) axis. The stimulation of CB1Rs in the preoptic nuclei of the hypothalamus reduces core body temperature, which may partially explain the symptomatic relief associated with hot baths [18].

As for the minimum duration of cannabis use required for symptom onset, it remains undefined. According to the most used diagnostic criteria, the minimum period of use should be one year. However, a 2020 study by Lonsdale H at al. focusing on the pediatric population reported symptom onset before one year of use, suggesting that reducing time to three months may be a more reasonable criterion [6]. In the case series of 98 patients reported by Simonetto et al., the duration of cannabis use before symptoms onset varied from 4 months to 27 years and the majority of them developed symptoms within 1 to 5 years of cannabis use [5].

### 3.3. Clinical Presentation

CHS is a disorder characterized by recurrent episodes of severe nausea and vomiting in individuals with chronic and heavy cannabis use.

Patients with CHS frequently present to emergency departments multiple times with similar symptoms, often undergoing extensive diagnostic tests and invasive procedures without achieving a definitive diagnosis or treatment plan [19].

**Figure 1:**
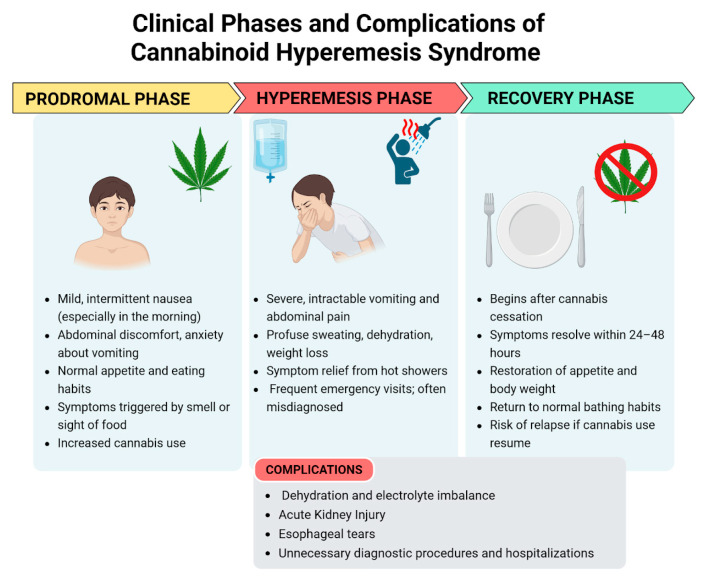
Clinical phases and complications of cannabinoid hyperemesis syndrome—created in BioRender. Pietrantoni, C. (2025) https://BioRender.com/bq3w7xu (accessed on 1 July 2025).

The clinical presentation of CHS can be categorized into three phases: the prodromal phase, the hyperemesis phase, and the recovery phase [1]. The clinical presentation is shown in Figure 1.

#### 3.3.1. Prodromal Phase

This phase is marked by mild symptoms such as morning nausea and abdominal discomfort [1]. It may begin months or even years prior to the onset of the hyperemesis phase. During this stage, patients commonly experience nausea, abdominal discomfort, and anxiety about vomiting. Nausea can be triggered by the sight or smell of food. These symptoms typically occur on one or more days per week and are more pronounced in the morning. Despite these symptoms, patients generally maintain a normal appetite and eating patterns, resulting in minimal weight changes. Patients may unknowingly increase their cannabis consumption to alleviate these symptoms, unaware that cannabis is the underlying cause of their discomfort [15].

#### 3.3.2. Hyperemesis Phase

This is the acute phase of CHS. The proportion of patients who transition from the prodromal phase to the hyperemesis phase is uncertain. This phase is defined by paroxysmal episodes of intractable nausea, vomiting, and abdominal pain which do not respond to various anti-emetic treatments. These episodic bouts of vomiting can be debilitating, and patients often experience additional symptoms such as sweating, colicky abdominal pain, and increased thirst. Vomiting can be so severe as to cause electrolyte imbalances, weight loss, and acute kidney injury [6]. Additionally, prolonged vomiting can lead to esophageal tears (Mallory–Weiss syndrome and Boerhaave syndrome) and rupture [20]. As a result, many patients visit the emergency department due to dehydration [15]. This phase typically lasts up to 48 h, although its duration can vary. It generally resolves with supportive care, which primarily includes fluid replacement and maintenance, along with anti-emetic medications.

During this phase, many patients take a hot shower to experience temporary relief from nausea, vomiting, and abdominal pain [5,21]. This behavior was thought to be so specific that it is occasionally utilized as a diagnostic criteria for CHS but its specificity was challenged in a recent comparative study, which found that 48% of patients with CVS, who did not use cannabis, experienced symptom relief with hot baths or showers, compared to 72% of those who used cannabis. Additionally, this hot water bathing behavior has been documented in preadolescents and adolescents with no history of cannabis exposure [3].

#### 3.3.3. Recovery Phase

The recovery phase begins with the cessation of cannabis use, with symptoms relief within 24–48 h. This phase is characterized by a return to relative well-being, the resumption of normal eating habits, the restoration of body weight, and regular bathing patterns [1]. It may last for days, weeks, or months depending on the cannabis use: if cannabis use is resumed at any point during this period, symptoms may return [15].

### 3.4. Diagnosis

In the absence of specific laboratory tests, diagnosis relies on the recognition of distinct clinical features. Delay in diagnosis can lead to considerable emotional, personal, and social distress for the family, and to an unnecessary financial burden on the healthcare system [15].

**Figure 2:**
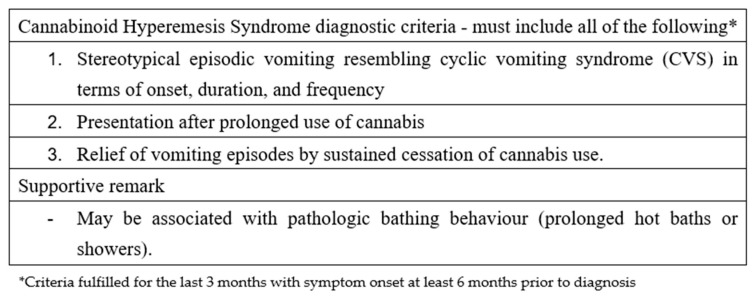
Roma IV criteria regarding cannabinoid hyperemesis syndrome.

CHS should be suspected in all patients presenting with intractable vomiting, a positive urine test for THC, and no other identifiable causes for vomiting [20]. Obtaining a detailed toxicological history can be challenging; therefore, testing for THC in urine should be considered in adolescents presenting with idiopathic vomiting characterized by a chronic or cyclical pattern. Over the years, various criteria have been proposed for diagnosing CHS; the most widely used in adults are the Rome IV criteria, as shown in Figure 2.

Among these criteria, the complete resolution of symptoms following a prolonged period of cannabis abstinence is required, which implies that no patient can be diagnosed during the acute phase of presentation. Furthermore, the required duration of this abstinence period is not clearly defined. These limitations have led to criticism of the Rome IV criteria, which are considered more useful for research than for clinical practice [22]. Consequently, there is a need for more practical criteria suitable for clinical settings. Such criteria were proposed in 2020 by Lonsdale et al., based on a 10-year case series [6]. These criteria are divided into major and minor categories.

The major criteria, reported in all patients in the case series, include the following:

- Regular cannabis use for at least 3 months;
- Onset or worsening of episodic nausea and vomiting resembling CVS after the initiation of regular cannabis use;
- Absence of other underlying medical conditions that could explain the symptoms, confirmed through appropriate negative investigations.

Supportive criteria include the following:

- Symptom relief with hot baths or showers;
- Weight loss;
- Abdominal pain;
- Altered bowel habits.

In differential diagnosis, it is essential to consider CVS, where cannabis use may be part of a self-treating behavior. Assessing the temporal relationship between cannabis use and symptom onset can aid in distinguishing CHS from CVS [22].

It is important to note that there is no universal consensus on whether CHS and CVS are distinct entities. CHS may instead be considered a subtype of CVS, where cannabis use acts as a trigger for the syndrome’s development in genetically predisposed individuals [3].

Other differential diagnoses include the following:

- Gastrointestinal conditions such as abdominal migraine, celiac disease, eosinophilic esophagitis, food sensitivities, gastroesophageal reflux disease, liver disease, pancreatitis, appendicitis, and anatomical obstruction such as malrotation or volvulus;
- Metabolic conditions such as mitochondrial dysfunctions;
- Neurological and psychiatric conditions such as migraines, intracranial masses, autonomic dysfunction, epilepsy, drug toxicity, anxiety, bulimia, and psychogenic vomiting;
- Endocrinological conditions [15].

### 3.5. Management and Treatment

The definitive treatment for CHS is the cessation of cannabis use. Regarding its management, there is no high-grade evidence in the literature for the effective treatment of CHS in children and adolescents [20]. In the acute phase, it is essential to provide rehydration with intravenous fluids, correct electrolyte imbalances, and use anti-emetic medications [1].

However, anti-emetic medications’ efficacy appears to be limited, as only one third of patients seem to experience relief; therefore, alternative therapies have been proposed for symptom management, including the use of benzodiazepines, haloperidol, and capsaicin cream. This observation may serve as an early indicator for the diagnosis of CHS [20].

Benzodiazepines, in particular lorazepam and clonazepam [20], are the most used drugs for managing CHS, both for their anti-emetic effects and support to cannabis withdrawal symptoms. The treatment duration should not exceed two weeks. A single intravenous dose of 1 mg of lorazepam has been found to halt emesis, with additional doses up to 1 mg every 6 to 8 h for the sustained cessation of vomiting [23].

Regarding haloperidol, although there is substantial supporting literature for its use in adult patients, data on the pediatric population are limited. Haloperidol exerts its pharmacological effect by antagonizing dopamine-2 (D2) receptors in the central nervous system. The HaVOC study (Intravenous Haloperidol Versus Ondansetron for Cannabis Hyperemesis Syndrome) demonstrated that intravenous haloperidol, administered at a dose of 0.05 mg/kg, was more effective than 8 mg of ondansetron in alleviating both abdominal pain and nausea in CHS patients presenting with active emesis. No extrapyramidal side effects were observed [24].

Topical capsaicin, applied to the abdomen, may help alleviate symptoms by activating TRPV1 receptors, which play a role in thermoregulation and endocannabinoid-induced pain relief. This mechanism is comparable to the effects of hot showers. Capsaicin cream is used in varying concentrations, with 0.075% being the most applied, followed by 0.025% and 0.1%. When applied to the abdomen, efficacy is typically observed within 20 to 30 min [21].

While managing esophagitis and gastritis resulting from repeated vomiting may justify adjunctive therapy with a proton pump inhibitor (PPI), their inclusion in anti-emetic combination regimens has not demonstrated any significant benefit in resolving CHS symptoms [23].

### 3.6. Prognosis

The only effective long-term treatment for CHS appears to be the cessation of cannabis use. However, the available data remain incomplete and require further standardization [1,6]. It is important to emphasize that patients may struggle to accept that cannabis is the cause of their symptoms, making cessation challenging [20].

CHS often presents comorbidly with attention deficit hyperactivity disorder, anxiety, and depression, and cannabis use is sometimes employed for the self-management of anxiety. Therefore, the treatment of CHS in the pediatric population may require a multidisciplinary approach to manage anxious and depressive symptoms in order to support cannabis cessation [1].

## 4. Discussion

Despite the growing recognition of cannabinoid hyperemesis syndrome (CHS), substantial uncertainties persist regarding its identification and management in pediatric populations. Most available data come from individual case reports or small case series, making it challenging to fully characterize the disease. Establishing a clear temporal correlation between cannabis use and symptom onset in adolescents remains particularly difficult. The minimum duration of cannabis exposure necessary to trigger symptoms is not well defined, and existing diagnostic criteria—such as the Rome IV criteria, which suggest a minimum of one year of cannabis use—may not be appropriate for younger patients. Emerging evidence indicates that a shorter exposure period, such as three months, may be more relevant in this age group. Moreover, the stigma surrounding substance use often leads to underreporting, making it difficult to obtain reliable histories [6]. The diagnostic process is further complicated by the absence of specific laboratory markers and the overlap of CHS symptoms with other gastrointestinal and functional disorders.

To date, the most widely used and recommended criteria for the diagnosis of CHS remain the Rome IV criteria, as they were developed through expert consensus. However, these criteria have limitations when applied to adolescents, particularly given the variability in cannabis use patterns and the clinical presentation of CHS in this population. The aim of this review was to present the current diagnostic frameworks, highlighting their limitations and discussing emerging proposals that may be more suitable for pediatric settings. We hope that future expert consensus efforts will lead to the development of more refined, age-appropriate diagnostic criteria that improve the recognition and management of CHS in adolescents.

Currently, no high-quality evidence supports specific acute phase treatments tailored to pediatric patients, and the only definitive intervention—cannabis cessation—lacks standardized strategies. Pediatric-specific clinical guidelines should include age-adapted diagnostic criteria, structured approaches to differential diagnosis, multidisciplinary management protocols that address both somatic and psychiatric dimensions, and comprehensive interventions to support cannabis cessation in adolescents.

## 5. Conclusions

CHS is a complex disorder that requires further research, particularly regarding its presentation and management in pediatric populations. While cannabis use continues to rise among adolescents, the true prevalence of CHS in this group remains unclear, and the diagnosis is often delayed due to its symptom overlap with other conditions. Early recognition is essential to avoid unnecessary diagnostic procedures and reduce healthcare burden. While cannabis cessation is the cornerstone of treatment, supporting adolescents through this process—especially those with psychiatric comorbidities such as anxiety and depression—requires a comprehensive, multidisciplinary approach. 

In light of these diagnostic challenges, emerging technologies such as artificial intelligence (AI) may offer valuable support. AI may offer valuable support by enhancing early detection. By analyzing electronic health records to identify patterns such as cyclic vomiting, chronic cannabis use, and symptom relief with hot showers, AI-integrated clinical decision support systems could help prompt the earlier consideration of CHS. Moreover, AI-based predictive models may assist in identifying adolescents at risk by analyzing behavioral and clinical data.

Given the increasing rates of cannabis use among adolescents, ongoing research is necessary to better define the clinical features and to standardize diagnostic criteria and treatment options for CHS in this vulnerable population.

## Data Availability

No new data were created or analyzed in this study.
